# Miscellaneous

**Published:** 1866-06

**Authors:** 


### SELECTED ARTICLES.

### MISCELLANEOUS.

*Nitro-Glycerine.*—The terribly explosive properties of this dangerous substance have been illustrated by a painful coincidence of events in San Francisco and Aspinwall, by which many lives and much property have been destroyed. It is strange that similar accidents have not previously occurred in the mining districts of the Pacific coast. Nitro-glycerine, or glonoine, as it is sometimes called in medicine, is prepared by allowing glycerine to fall drop by drop into a mixture of equal parts of nitric acid and oil of vitriol, care being taken to prevent the temperature from rising too high. A heavy, oily-looking liquid collects at the bottom of the vessel, which has a sweetish aromatic taste, and is a dangerous poison. A single drop placed upon the tongue produces a severe pain in the head for hours. Its formula is $C_6\ H_6\ (NO_4)\ _2O_6$, or for two equivalents of hydrogen in the glycerine two of peroxide of nitrogen are substituted. A drop placed upon paper and struck on an anvil produces a powerful concussion. Under circumstances little understood the friction of its particles among themselves even when frozen, or contact with some foreign substance, is sufficient to produce such effects as those below given. Its manufacture or storage in some parts of Europe is forbidden, and the Mayor of New York has acted wisely in causing the removal of all found in that city.

The following particulars of the disaster are from the *Panama Star and Herald:*

"Nearly, if not all the local freight of the European had been delivered, when about 7 o'clock in the morning of the 3d, a terrific explosion occurred on board, which tore away the upper parts of the ship and blew several large plates off the side. The wharf at which the ship was unloading, and which was some four hundred feet long, was literally torn to pieces; the superstructure was completely demolished to within a hundred feet of the freight house, and hardly a plank remained in the entire length of the structure that was not wrenched from its fastenings.

"Immediately in front of where the vessel lay, a gap was cut through the wharf, piles, planking, etc., all disappearing. The ship and wharf both caught fire, and the latter was saved from entire destruction only by the exertions of several citizens, who got the fire engine to work, and after a few hours extinguished the flames, regardless of the risk they incurred from another explosion of the burning ship. The Panama Railroad Company's splendid freight house is left a pile of ruins. The force of air caused by the concussion seems to have raised the roof—which was constructed of iron and slate—upwards a few feet, its own weight bringing it down with immense force into the building, and carrying with it both the end walls, leaving the house, excepting the side walls, which appear but little if at all injured, a mass of ruins. * * * * *

"The most awful part of the catastrophe was the dreadful loss of life and suffering attending it. Of the number killed and missing it is impossible to give a correct estimate; but from present data the number may be safely put down at fifty, and is, we fear, more likely to prove over this number than under it. Of the forty-one men comprising the crew of the European, nine have been killed and twelve are missing.

"The scene in Aspinwall after the first explosion cannot be described—it was harrowing in the extreme. Whilst the ruins gave an air of desolation to the place, the mangled and lacerated bodies or pieces of bodies, to be met with in every direction for a great distance around the ruin of the disaster, were heart-rending, and the suffering of the poor mortals crushed and bruised, in whom life was not extinct, was really dreadful.

"The amount of damage caused by the explosion is roughly estimated at $1,000,000, which is about the lowest figure at which it can be placed."—*Boston Med. and Surg. Journal.*

---

*Endermic Poisoning by Belladonna.*—The application of belladonna to the breast for the relief of painful distention of the organs, especially after sudden weaning, is often resorted to, and with advantage. Where there is an abrasion of the skin, however, this practice, it should be known, is not devoid of danger. A case of poisoning, under such circumstances, is recorded in a recent number of the *Lancet*, November 11, 1865. —*Journal of Medicine.*

---

*Cancer* is nearly three times more fatal among women than among men. Of eight thousand seven hundred deaths from cancer in women, about three thousand, or more than one-third of all cases of cancer in the females, were instances of cancer of the uterus.—*Simpson.*

